# Katanin: A Sword Cutting Microtubules for Cellular, Developmental, and Physiological Purposes

**DOI:** 10.3389/fpls.2017.01982

**Published:** 2017-11-21

**Authors:** Ivan Luptovčiak, George Komis, Tomáš Takáč, Miroslav Ovečka, Jozef Šamaj

**Affiliations:** Department of Cell Biology, Centre of the Region Haná for Biotechnological and Agricultural Research, Faculty of Science, Palacký University, Olomouc, Czechia

**Keywords:** cytoskeleton, katanin, microtubules, morphogenesis, plant development, cell division, cell growth, hormone

## Abstract

KATANIN is a well-studied microtubule severing protein affecting microtubule organization and dynamic properties in higher plants. By regulating mitotic and cytokinetic and cortical microtubule arrays it is involved in the progression of cell division and cell division plane orientation. KATANIN is also involved in cell elongation and morphogenesis during plant growth. In this way KATANIN plays critical roles in diverse plant developmental processes including the development of pollen, embryo, seed, meristem, root, hypocotyl, cotyledon, leaf, shoot, and silique. KATANIN-dependent microtubule regulation seems to be under the control of plant hormones. This minireview provides an overview on available *KATANIN* mutants and discusses advances in our understanding of KATANIN biological roles in plants.

## Introduction

KATANIN is a conserved AAA ATPase protein complex severing microtubules and it was discovered in *Xenopus laevis* (McNally and Vale, 1993). KATANIN is a heterodimer formed from catalytic p60 and regulatory p80 subunits while its severing activity requires hexamerization on the microtubule surface (Hartman and Vale, 1999; Stoppin-Mellet et al., 2007). Catalytic p60 subunit represents a microtubule-stimulated ATPase that requires ATP hydrolysis to sever microtubules. The exact timing of severing is not well-understood since KATANIN likely moves along the microtubule before severing it (Eckert et al., 2012). Factors that may specify the KATANIN-mediated microtubule severing include microtubule lattice defects (Davis et al., 2002; Díaz-Valencia et al., 2011; Jiang et al., 2017), tubulin post-translational modifications (Sudo and Baas, 2010), and the occurrence of free tubulin dimers which may inhibit KATANIN activity through their carboxyl terminal tails (Bailey et al., 2015). The power stroke leading to microtubule severing is depending on a nucleotide-based transition of KATANIN oligomers between an open spiral structure (all p60 protomer nucleotide binding sites are occupied by ATP) and a closed, nucleotide devoid, ring structure that requires ATP hydrolysis (Zehr et al., 2017). Regulatory p80 subunit is a WD40-containing protein which is stimulating the microtubule severing activity of the p60 subunit and in animal cells is thought to mediate KATANIN targeting to specific sites such as the centrosome while potentiating microtubule binding (e.g., McNally and Vale, 1993; Hartman et al., 1998; McNally et al., 2000). The plant p60 subunit suffices for microtubule severing (Stoppin-Mellet et al., 2002), but the targeting of Arabidopsis KATANIN to specific microtubule severing sites (see later) is mediated by four different p80 subunits (Wang et al., 2017). Studies in rice showed that the overexpression of a p80 ortholog (OsKTN80a) relates to cell elongation and cell division defects (Wan et al., 2014). In many animal models studied so far, KATANIN activity and localization is also depending on its interactions with TUBULINS and other proteins.

To simplify the text, KATANIN will be used here for the Arabidopsis KATANIN p60. KATANIN deserves a special attention in this review for three reasons. First, ever since its discovery and functional characterization, (McClinton et al., 2001; Stoppin-Mellet et al., 2002) it is a dominant microtubule regulator in plants as evident by studies on various *KATANIN* mutants with rather severe phenotypes (see later). However, it is worth to note that there are very scarce studies on plant homologs of other microtubule severing AAA-ATPases such as FIDGETIN (Girard et al., 2015) and SPASTIN (Gardiner, 2014) Second, many studies described KATANIN key roles in the organization of cortical microtubule arrays (Stoppin-Mellet et al., 2006; Wightman and Turner, 2007; Nakamura et al., 2010; Lindeboom et al., 2013; Wightman et al., 2013; Zhang et al., 2013; Komis et al., 2017). Third, KATANIN is involved in the regulation of cell division plane orientation (Panteris et al., 2011; Panteris and Adamakis, 2012; Komis et al., 2017) and in the mitotic and cytokinetic progression (Komis et al., 2017).

## Characterization of arabidopsis KATANIN

The first plant homolog of animal KATANIN p60 was identified in *Arabidopsis thaliana*. Since several independent groups studied functions of the Arabidopsis KATANIN p60 simultaneously, the respective gene was identified under diverse names such as *BOTERO1* (Bichet et al., 2001), *AtKTN1* (Burk et al., 2001), *AtKSS* (McClinton et al., 2001), *Atp60* (Stoppin-Mellet et al., 2002), *ECTOPIC ROOT HAIR 3* (*ERH3*; Webb et al., 2002), *FRAGILE FIBER 2* (*FRA2*, Burk et al., 2001), or *LUE1* (Bouquin et al., 2003). KATANIN is composed of 523 amino acid residues with a calculated molecular mass of 57.27 kDa. An *in vitro* ATP-dependent microtubule severing activity was described for recombinant plant KATANIN (Burk and Ye, 2002; Stoppin-Mellet et al., 2002). The Arabidopsis genome contains a single *KATANIN p60* and four *KATANIN p80* homologs (GenBank accession numbers: AAB71 474, CAC08 339, AAD4 999, and BAB09 559; Bouquin et al., 2003; Keech et al., 2010). All these p80 homologs contain N-terminal WD40 domains, which is involved in the subcellular targeting of KATANIN.

## Cellular functions of plant KATANIN

Cellular functions of plant KATANIN have been a matter of intensive research during the past decade. It became apparent that KATANIN is essential to break the initial isotropy of cortical microtubule organization and bias their parallel organization during conditional or developmental establishment of cell growth directionality. KATANIN plays a central role in the coordination of shoot apical meristem growth and emergence of leaf primordia in relation to compressive mechanical forces (Uyttewaal et al., 2012) and auxin signaling (Sassi et al., 2014). It is also involved in blue light- or auxin-induced hypocotyl cell elongation (Lindeboom et al., 2013; Lin et al., 2013).

In this regard, KATANIN was shown to sever emergent, γ-TUBULIN and AUGMIN-nucleated microtubules, which branch-off from pre-existing microtubules (Nakamura et al., 2010) as well as microtubules that cross each other during their elongation (Wightman and Turner, 2007, 2008; Zhang et al., 2016). Such cross-severing ability of KATANIN is considered to be important for the reorientation of cortical microtubules by blue light or ethylene (Soga et al., 2010a; Lindeboom et al., 2013) since it promotes disassembly of microtubules with unfavorable orientation. A recent modeling study uncovered the possibility that KATANIN-mediated severing at microtubule crossovers should only occur under permissive angles of encounter, below which microtubule bundling is more likely (Deinum et al., 2017). Moreover, KATANIN activity might support microtubule bundling (Stoppin-Mellet et al., 2006). There are also other microtubule binding proteins, e.g., TORTIFOLIA1 (SPIRAL2), which can regulate KATANIN severing activity (Buschmann et al., 2004; Wightman et al., 2013).

## The role of KATANIN in plant mitosis and cell division plane orientation

Some studies proposed the role of KATANIN in microtubule organization within preprophase band (PPB; Panteris et al., 2011; Komis et al., 2017) but showed conflicting results regarding the role of KATANIN in mitotic spindle formation. In this respect *fra2, lue1, bot1*, and *ktn1-2* mutants possessed pronounced multipolar prophase spindles (Panteris et al., 2011; Panteris and Adamakis, 2012), although a previous study using tubulin immunolabeling failed to show spindle defects in *fra2* mutant (Burk et al., 2001). The latter results were recently corroborated with live cell imaging of dividing *ktn1-2* cells which showed normal spindle formation but marked delay in mitotic progression and deregulation of spindle positioning throughout mitosis (Komis et al., 2017). Microtubule organization of the phragmoplast was also affected (Panteris et al., 2011; Komis et al., 2017), while its centrifugal expansion was delayed (Komis et al., 2017). In the context of cell division plane orientation some studies showed disorganized cell files in roots of various *KATANIN* mutants (Bichet et al., 2001; Webb et al., 2002; Panteris et al., 2011). Although it was suggested that spindle multipolarity in *KATANIN* mutants might define the plane of phragmoplast expansion (Panteris et al., 2011), it was later shown that it rather followed the plane of the aberrant PPB (Komis et al., 2017). The connection of PPB to cell division plane orientation was very recently revisited revealing that PPB is involved in mitotic spindle positioning (Schaefer et al., 2017). In this respect, our work on the *ktn1-2* mutant showed aberrant PPB formation and uncontrolled spindle rotational motions during cell division (Komis et al., 2017). Although these results are preliminary, they suggest a role of KATANIN in spindle—cell cortex interactions which are co-aligning the spindle equator with the PPB-determined cell division zone (Smertenko et al., 2017) and define the geometry of cell plate expansion later during cytokinesis.

On the other hand, spindle assembly was not visibly affected and it is a pending question whether the localization of KATANIN in Arabidopsis mitotic spindles (Panteris et al., 2011) regulates spindle formation and shape or spindle sizing as it was previously reported (McNally et al., 2006; Loughlin et al., 2011; Panteris et al., 2011).

## Identification of developmental roles of KATANIN using mutants

There have been many *KATANIN* mutants identified during the past years and for many of them information on the nature of the mutation is missing (Table 1). Some mutants are predicted to lack KATANIN or encode truncated versions while others presumably affect some particular protein domain (e.g., the AAA-ATPase domain as in the case of *erh3* alleles; Table 1) but with unknown consequences for the functionality of the protein produced. On the other hand, the inducible overexpression of KATANIN was shown to cause heavy fragmentation of cortical microtubules while favoring microtubule bundling in Arabidopsis pavement cells (Stoppin-Mellet et al., 2006). Table 1 also provides comparative information related to phenotypes of diverse *KATANIN* mutants.

**Table 1:** Genetic characteristics of Arabidopsis *KATANIN* mutants and *in silico* prediction of modifications in amino acid sequences.

**Allele**	**Ecotype**	**Mutagen**	**Genomic sequence**	**Coding DNA sequence**	**Exon**	**Codon modification**	**Amino acid modification**	**Position in domain**	**References**
*bot1-1*	Col-0	EMS	n.a.	n.a.	n.a.	n.a.	n.a.	n.a.	Bichet et al., 2001
*bot1-2*	C-24	Tnt1	n.a.	n.a.	n.a.	n.a.	n.a.	n.a.	Lucas et al., 1995; Bichet et al., 2001
*bot1-3*	Col-0	EMS	n.a.	n.a.	n.a.	n.a.	n.a.	n.a.	Bichet et al., 2001
*bot1-4*	Col-0	EMS	n.a.	n.a.	n.a.	n.a.	n.a.	n.a.	Bichet et al., 2001
*bot1-5*	Ler-0	EMS	n.a.	n.a.	n.a.	n.a.	n.a.	n.a.	Reed et al., 1998
*bot1-7*	Ws	T-DNA	32–50 bp del	32–50 bp del	1. exon	TTA-TAG	L17-stop	p80 interacting domain	Bichet et al., 2001; Uyttewaal et al., 2012
*bot1-8*	Ws	T-DNA	n.a.	n.a.	n.a.	n.a.	n.a.	n.a.	Bichet et al., 2001
*erh3-1*	Col-0	EMS	C1863T	C1057T	5. exon	CAT-TAT	H353Y	AAA domain	Schneider et al., 1997; Webb et al., 2002
*erh3-2*	Col-0	EMS	G1551A	G820A	4. exon	GGA-AGA	G274R	AAA domain, close to Walker A	Webb et al., 2002
*erh3-3*	Col-0	EMS	C1648T	C917T	4. exon	GCT-GTT	A306V	AAA domain	Webb et al., 2002
*fra2*	Col-0	EMS	A2329 del	A1349 del	7. exon	CTG-TGA	L452-stop	Vps domain is missing	Burk et al., 2001
*frc2-1*	Col-0	EMS	n.a.	n.a.	n.a.	n.a.	n.a.	n.a.	Luo and Oppenheimer, 1999
*frc2-2*	Col-0	EMS	n.a.	n.a.	n.a.	n.a.	n.a.	n.a.	Luo and Oppenheimer, 1999
*frc2-3*	RLD	Fast neutrons	n.a.	n.a.	n.a.	n.a.	n.a.	n.a.	Luo and Oppenheimer, 1999
*ktn1-1*	n.a.	n.a.	1584A ins	854A ins	4. exon	GAG-TGA	E295-stop	AAA domain is missing	Nakamura et al., 2010
*ktn1-2*	Col-0	T-DNA	n.a.	n.a.	5. exon	n.a.	n.a.	AAA domain	Nakamura et al., 2010
*ktn1-3*	Col-0	EMS	C1335T	C773T	3. exon	CCT-CTT	P258L	rfcL domain	Lin et al., 2013
*ktn1-4*	n.a.	EMS	C2359T	C1379T	7. exon	TCA-TTA	S460L	between AAA and Vps domains	Lin et al., 2013
*ktn1-5*	n.a.	T-DNA	n.a.	n.a.	5. exon	n.a.	n.a.	AAA domain	Lin et al., 2013
*ktn1-6*	Col-0	EMS	C1657T	C926T	4. exon	GCT-GTT	A309V	AAA domain is cut	Ren et al., 2017
*lue1*	Col-0	n.a.	G1988A	G1182A	5. exon	TGG-TGA	W394-stop	AAA domain, Vps domain is missing	Meier et al., 2001; Bouquin et al., 2003
*mad5*	n.a.	n.a.	G3A	G3A	1. exon	ATG-ATA	M1I	n.a.	Brodersen et al., 2008

*del, deletion; ins, insertion; EMS, ethyl methanesulfonate; Tnt1, retrotransposon Tnt1 mediated mutation. n.a., data not available; Domain characterization of AtKTN1 was predicted based on sequence homology with human katanin (Gosh et al., 2012)*.

The first described Arabidopsis mutant of *KATANIN* in the locus named *BOTERO1* (*BOT1*) was *bot1-1*. It is an EMS (ethyl methane sulfonate) mutant in the genetic background of Col-0 ecotype. Seven alleles have been described in this locus: *bot1-1* to *bot1-5, bot1-7*, and *bot1-8* (Tables 1, 2; Bichet et al., 2001). These mutants exhibit radially swollen cells with isotropic cortical microtubule arrays, suggesting a specific role of *BOT1* in the organization of cortical microtubules during cell elongation. Thus, *botero1* mutant has round and thick leaves and thick stems. Hypocotyls are shorter and thicker, inflorescence stems have shorter internodes and shorter anthers might cause reduced fertility. Cortical microtubules of leaf primordia show delayed reorientation responses during mechanical stimulation (Uyttewaal et al., 2012). However, the mutation has no effect on tip growth of root hairs and pollen tubes, and on trichome morphogenesis (Bichet et al., 2001). During mitosis, PPB microtubules in *bot1* root cells exhibit malorganized and defective perinuclear formation while the cytokinetic phragmoplast shows aberrant microtubule organization (Panteris and Adamakis, 2012).

**Table 2:** Overview of (sub)cellular and developmental defects in plant *KATANIN* mutants.

**Locus (species)**	**Shortcut of locus**	**Allele (mutant)**	**Defects of microtubules and cell growth**	**Defects in vegetative organs**	**Defects in reproductive organs**	**Reference**
*BOTERO1 (A. thaliana)*	*BOT1*	*bot1-1*	Anisotropic growth in all non-tip-growing cell types, reduced cell length, short and thick cells, more compact organs	Round-shaped and thick leaves, inflorescence stems with short internodes, delayed senescence of mutant plants	Stubby flower organs, mechanically sterile flowers as a result of reduced anther length	Bichet et al., 2001
*DWARF AND GLADIUS LEAF 1 (O. sativa)*	*DGL1*	*dgl1-1, dgl1-2, dgl1-3*		Disturbed leaf blade morphology with short and round shaped leaves	Disturbed floral organ development	Komorisono et al., 2005
*ECTOPIC ROOT HAIR 3 (A. thaliana)*	*ERH3*	*erh3-1, erh3-2, erh3-3*	Disorganized cortical microtubules, abnormal positioning of cell walls	Ectopic root hairs and hairless cells, root radial swelling, stem and inflorescence stem fragility		Schneider et al., 1997; Webb et al., 2002
*FRAGILE FIBER 2 (A. thaliana)*	*FRA2/AtKTN1*	*fra2*	Disorganized cortical microtubules and reduced cellulose content, increased cell width, impaired cell elongation	Short and radially swollen roots, ectopic root hairs, round and compact leaves in the rosette with small blades, stem and inflorescence stem fragility, reduced size of inflorescence stem, disoriented cell divisions in the pro-embryo, abnormally shaped hypophysis, most trichomes on leaves with two branches instead of three branches	Short and thick sepals, petals, pistils and anthers; siliques with unfertilized ovules, reduced number of developing seeds, reduced carpel size and abnormal carpel junctions, malformed ovule development, anther lobes irregularly developed containing variable pollen viability	Burk et al., 2001; Luptovciak et al., 2017
*FURCA2 (A. thaliana)*	*FRC2*	*frc2-1, frc2-2, frc2-3*		Exhibit about 60% of the trichomes with two branches instead of three branches	Decreased fertility compared to wild-type, premature extension of the pistil from the unopened flower	Luo and Oppenheimer, 1999
*KATANIN 1 (A. thaliana)*	*KTN1*	*ktn1-2*	Random orientation of perinuclear microtubules and multipolar bundling, disorganized microtubules during cytokinesis in root cells, deregulation of spindle positioning throughout mitosis, defects in cell division plane orientation	Disoriented cell divisions in the pro-embryo, abnormally shaped hypophysis	Short and thick siliques with unfertilized ovules and reduced number of developing seeds, large and misshaped seeds, reduced carpel size and abnormal carpel junctions, malformed ovule development, anther lobes irregularly developed containing variable pollen viability	Nakamura et al., 2010; Komis et al., 2017; Luptovciak et al., 2017
*LUCIFERASE SUPER-EXPRESSOR 1 (A. thaliana)*	*LUE1/AtKSS*	*lue1*	Disorganized cortical microtubules, increased cell width, impaired cell elongation	Short and radially swollen roots with ectopic root hairs, shorter leaves, short and fragile stems, reduced number of secondary shoots, disoriented cell divisions in the pro-embryo, abnormally shaped hypophysis	reduced carpel size and abnormal carpel junctions, malformed ovule development, anther lobes irregularly developed containing variable pollen viability, shorter, and thicker siliques with unfertilized ovules and reduced number of developing seeds, round-shaped seeds, increased seed size	Meier et al., 2001; Bouquin et al., 2003; Luptovciak et al., 2017

**Figure 1:**
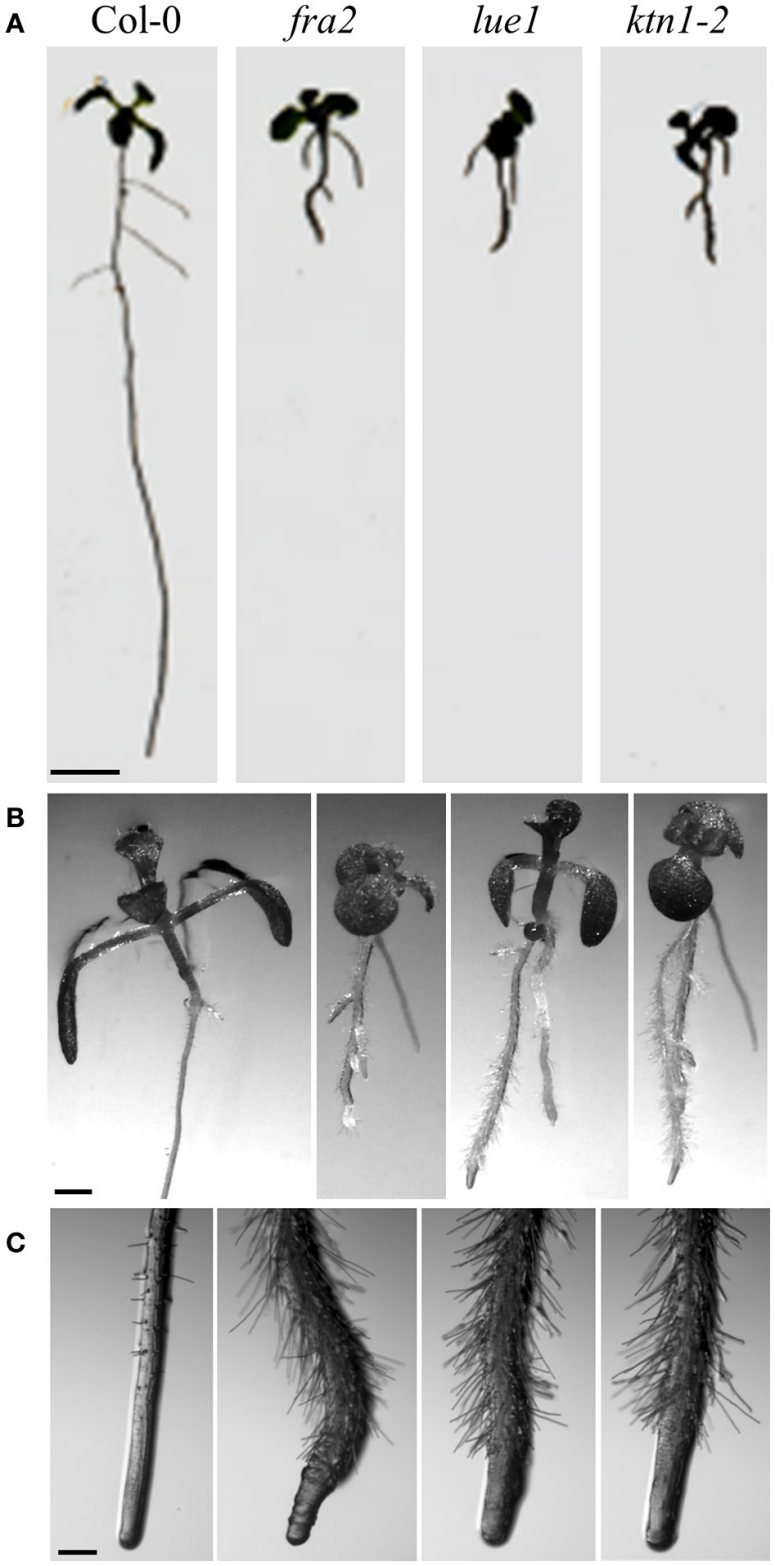
Morphological phenotypes of *KATANIN* mutants vs. Col-0 wild-type (7 days old seedlings). **(A)** Representative pictures of whole Col-0 seedlings and *KATANIN* mutants. Note much shorter roots in the mutants. **(B)** Detailed pictures of above ground seedling parts in Col-0 and *KATANIN* mutants. **(C)** Detailed pictures of primary root tips of Col-0 and *KATANIN* mutants. Note radial expansion of mutant roots. Scale bars = 5 mm **(A)**; 1 mm **(B)**; 250 μm **(C)**.

The mutant *fragile fiber 2* (*fra2*) is an EMS mutant (Tables 1, 2) which displays fragility of all organs and particularly of stems. This fragility is accompanied by reduced cellulose deposition, resulting in shorter and thinner fibers and increased fragility due to the lower mechanical resistance. Cell growth is aberrant in all organs of such mutant with pleiotropic phenotypes. As reported trichome morphogenesis is affected (Burk et al., 2001) unlike to what was observed for *botero* mutants (Bichet et al., 2001). The mutant displays dwarf phenotype (Figure 1). The size of inflorescence stem is strongly reduced and it is related to the decreased length of internodes, but not to their lower number. Rosette leaves are more round-shaped and have smaller blades. Sepals, petals, pistils, anthers, and siliques are shorter and thicker. Siliques bear unfertilized ovules and seed set is reduced. Ovule development is variably defective ranging from normal to severely malformed, suggesting an abnormal migration and positioning of nuclei and problematic polar nuclei fusion (Burk et al., 2001; Luptovciak et al., 2017). Most trichomes on *fra2* leaves have two instead of three branches (Burk et al., 2001).

Luo and Oppenheimer (1999) have identified mutants that show a reduction in trichome branch number from three branches to two and they named them *furca* (two-pronged fork in the Latin). One of these genes called *FURCA2* (*FRC2*) was later replaced in TAIR database as *BOT1* and *ERH3*. There are three *frc2* alleles: *frc2-1, frc2-2, frc2-3*, and about 60% of their trichomes show two branches. *frc2-1* and *frc2-3* have apparently decreased fertility compared to the wild-type plants which is caused by a premature extension of the pistil from the unopened flower before anthers mature.

Another set of EMS mutants is designated as *ectopic root hair 3* (*erh 3-1 to erh1-3*; Tables 1, 2) as they display disturbed root and root hair phenotypes. In this case, the authors studied root hair emergence and the developmental succession of trichoblasts and atrichoblasts in the root epidermis, rather than the process of root hair tip growth. All three *erh3* mutants have point mutations in the AAA-ATPase domain, however, it is not clear whether this can influence nucleotide occupancy and subsequently KATANIN oligomerization and severing. The most severe phenotypical defects have been observed in the *erh3-2* mutant where the root is the shortest compared to the wild type and appears radially swollen while root tissue patterning is disturbed (Webb et al., 2002).

The *lue1* mutation was originally isolated in the screening of mutants with altered expression of GA20-oxidase, and the gene was named *LUCIFERASE SUPER-EXPRESSOR1* (*LUE1*; Tables 1, 2). It is a recessive mutation displaying dwarf mutant phenotype achieving only 30% of the wild type size (Meier et al., 2001; Bouquin et al., 2003) (Figure 1). Cortical microtubules and cellulose microfibrils are abnormally arranged in the *lue1* mutant. In comparison to the wild type, *lue1* mutant has shorter leaves and inflorescences, while siliques contain 80% less seeds being bigger and more round (Luptovciak et al., 2017). This mutant also shows altered ethylene sensitivity leading to the improper formation of the hypocotyl hook and to decreased hypocotyl elongation (Bouquin et al., 2003).

**Figure 2:**
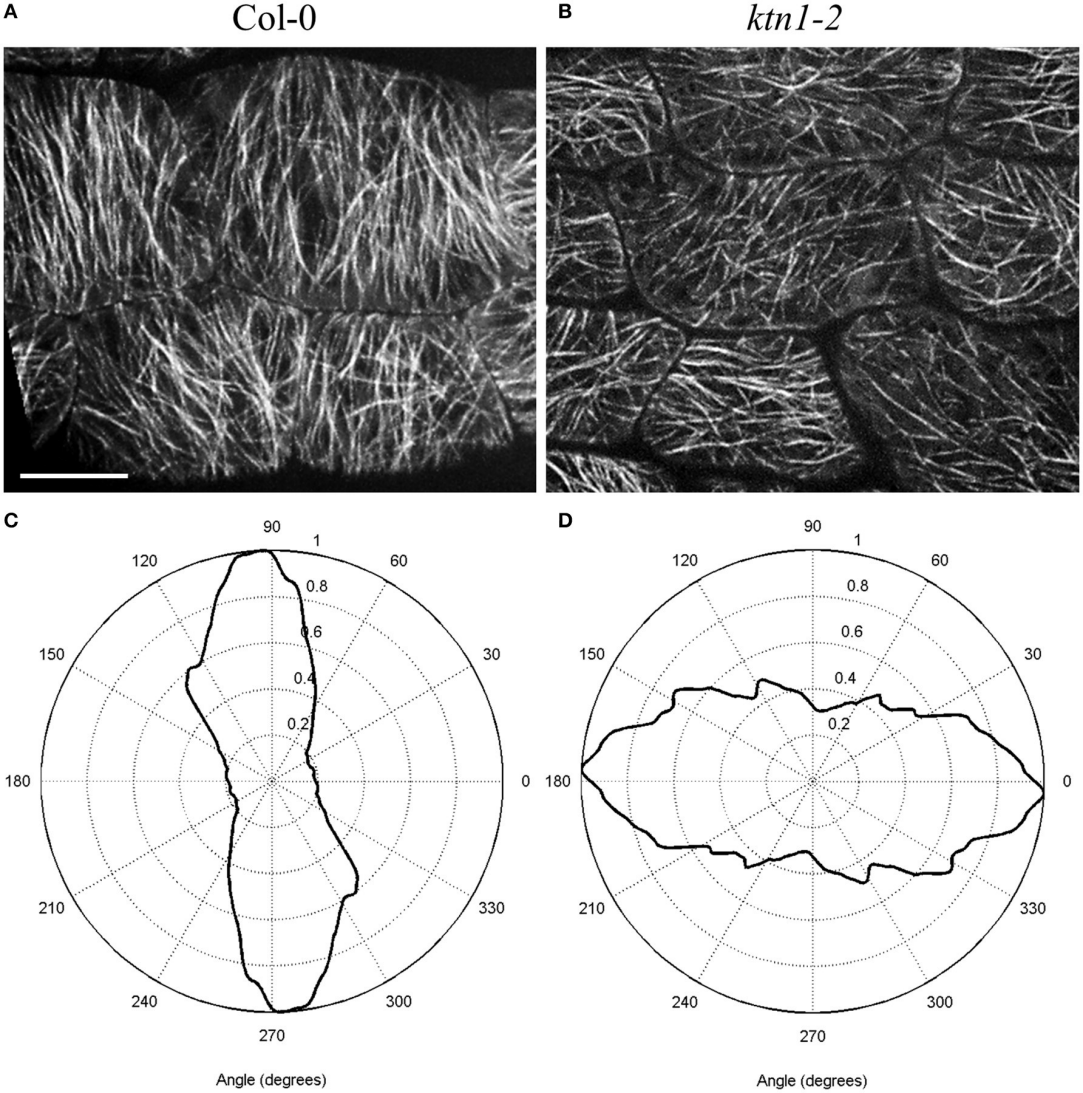
Differences in the microtubule organization and cell shape of *Arabidopsis thaliana* Col-0 and *ktn1-2* petiole epidermal cells carrying a GFP-TUA6 microtubule marker after spinning disk microscopy imaging. In differentiated cells, most cortical microtubules are transversal to the cell axis in Col-0 showing parallel placement to each other **(A)** resulting in relatively narrow angular distribution **(C)** whereas they appear more disorganized and randomly oriented in *ktn1-2* **(B)** exhibiting broader angular distribution pattern **(D)**. Scale bar = 10 μm.

Next, there are five *KATANIN 1* mutants named *ktn1*-*1* to *ktn1*-*5* with *ktn1-2 and ktn1-5* being T-DNA null mutants, and *ktn1-1, ktn1-3*, and *ktn1-4* being point mutants (Tables 1, 2; Nakamura et al., 2010; Lin et al., 2013). The *ktn1-2* mutant displays dwarf phenotype (Figure 1). Fertility is reduced in the null *ktn1-2* mutant, while aberrant orientations of cell divisions and abnormal formation of hypophysis affect embryogenesis (Luptovciak et al., 2017). In addition, many developmental phenotypes of *ktn* mutants are consistent with described phenotypes of other *KATANIN* mutants. For example, *ktn1-2* mutant shows defective organization of mitotic microtubule arrays, delay in mitotic progression and orientation defects of these arrays during cytokinesis (Panteris and Adamakis, 2012; Komis et al., 2017). Moreover, cortical microtubule organization and dynamics were also shown to be affected in the hypocotyl, petiole (Figure 2) and leaf epidermal cells (Lindeboom et al., 2013; Zhang et al., 2013; Komis et al., 2017).

The homolog of *KATANIN* in rice (*Oryza sativa*) is named *DWARF AND GLADIUS LEAF 1* (*DGL1*; Table 2). Three mutants, named *dgl1-1, dgl1-2*, and *dgl1-3* were identified and display similar traits like dwarfism, differentially disturbed floral organ development and leaf blade morphology with shorter and more round leaves. These mutants show minimal response to gibberellins and brassinosteroids, two hormones regulating the stem height (Komorisono et al., 2005).

A decade ago, Nagawa et al. (2006) created the KG12419 gene trap line with disrupted *KATANIN* gene and showing GUS staining under the control of *KTN* promoter. This line exhibited a high GUS activity in the procambium cells (elongated precursors of vascular cells) of various organs. These authors observed a significant shortening of procambium cells of higher order vein in young leaves as well as aberrant trichomes and defective root hair formation in the homozygous KG12419 line.

## Physiological functions of KATANIN

Studies on *KATANIN* mutants revealed that the altered microtubule severing has also a number of physiological consequences. First of all, KATANIN is a microtubule-interacting protein which can connect the external environment with microtubule regulation (Nakamura, 2015). The sensitivity of KATANIN to external stimuli is likely mediated by Rho GTPase signaling (Lin et al., 2013), however, other mechanisms cannot be excluded. External stimuli cause reorientation of microtubules followed by specific developmental reprogramming. Such mechanism is linking hormonal regulation to the cytoskeleton, because KATANIN is involved in gibberellic acid (GA) (Meier et al., 2001; Bouquin et al., 2003) ethylene (Soga et al., 2010a,b) and auxin responses (Chen et al., 2014).

Arabidopsis *KATANIN* mutants show defective microtubule reorientation in response to GA, ethylene and auxin (Bouquin et al., 2003; Komorisono et al., 2005; Soga et al., 2010a; Chen et al., 2014; Sassi et al., 2014). *KATANIN* transcript accumulation (by an unknown mechanism) is induced by these hormones (Bouquin et al., 2003; Soga et al., 2010a, eFP browser hormone series http://bbc.botany.utoronto.ca/efp). It is therefore supposed to control GA, ethylene, and auxin-dependent developmental processes requiring microtubule reorientation. Impaired responses of *KATANIN* mutants are likely connected to the altered synthesis and metabolism of hormones. This was demonstrated by increased mRNA levels of GA biosynthetic enzymes in rice mutant *dgl1* (Komorisono et al., 2005) and by changed abundances of proteins involved in GA, ethylene, auxin, and abscisic acid metabolism in *fra2* and *ktn1-2* mutants (Takáč et al., 2017). Thus, KATANIN might be a protein integrating multiple microtubule-dependent hormonal responses. Other proteins involved in hormonal signal integration are DELLA proteins (Achard et al., 2006; Santner and Estelle, 2009). DELLA proteins are suppressed by GA via their destabilization (Fu et al., 2002). Interestingly, DELLA proteins have been found to mediate microtubule reorientation during GA response. The mechanism is based on DELLA interaction with prefoldin complex, which is disrupted by DELLA degradation during GA response. Prefoldin complex is important for tubulin folding (Gu et al., 2008). When mislocalized to the nucleus it causes altered microtubule organization (Locascio et al., 2013). It was proposed that apart from tubulin monomer supply, an additional mechanism such as microtubule severing can be implicated in the final organization of microtubule arrays (Locascio et al., 2013). According to these reports, KATANIN might work in concert with DELLA-prefoldin complex. Nevertheless, a molecular interplay between DELLA and KATANIN remains to be confirmed. KATANIN likely is not required for DELLA degradation, since DELLA is destabilized in the rice *dgl1* mutant (Komorisono et al., 2005).

In addition to hormonal stimuli, KATANIN mediates the reorientation of cortical microtubules upon blue light exposure (Lindeboom et al., 2013). Under blue light, hypocotyl phototropism was connected to KATANIN severing activity at the microtubule crossovers, resulting in the formation of new microtubules with transversal orientation. KATANIN is regulated by phototropin receptor, as evidenced by cell biological analyses of *phot1phot2* double mutant (Lindeboom et al., 2013). In this context, KATANIN was identified by using yeast two hybrid assay as an interactor of the actin-binding kinesin-like protein KIN-14A (Bouquin et al., 2003; Suetsugu et al., 2012). This is a microtubule motor protein involved in blue light induced chloroplast movements (Suetsugu et al., 2010), where phototropins PHOT1 and PHOT2 play a crucial role (Luesse et al., 2010). Therefore, it is likely that interactions between KATANIN, phototropins and kinesin-like protein KIN-14A govern the blue light induced chloroplast movement. Microtubule severing is also required for plant responses to mechanical forces (Uyttewaal et al., 2012; Sampathkumar et al., 2014). This is essential for the control of cell shape, growth anisotropy and plant morphogenesis.

KATANIN is also important for translational miRNA-mediated repression, as revealed by genetic screen (Brodersen et al., 2008). The *Arabidopsis* mutant *mad5*, carrying G-to-A transition in the start codon of *KATANIN*, showed increased abundances of miRNA-targeted proteins. Similar results were obtained in *fra2* and *lue1* mutants, whose genetic background differs from that of *mad5* (Table 1). Defects in miRNA-mediated translational repression might suggest that some overabundant proteins in *fra2* and *lue1* mutants found in a recent proteomic study (Takáč et al., 2017) belong to miRNA-targeted proteins. Notably, *fra2* and *lue1* mutants also showed substantial alterations in the translational machinery, represented by changes in the abundance of ribosomal proteins (Takáč et al., 2017). The mRNA silencing machinery is closely connected to stress granules (Mollet et al., 2008). Stress granules are assembled during environmental stress and are sites of post-transcriptional mRNA processing and silencing (Anderson and Kedersha, 2008). KATANIN is implicated in stress granule formation (Gutierrez-Beltran et al., 2015) and mediates the abundance and the accumulation of Tudor staphylococcal nuclease proteins (TSN1 and 2) (Takáč et al., 2017). These proteins are important for stress-induced mRNA decapping in the stress granules and they modulate abiotic stress responses (Gutierrez-Beltran et al., 2015).

## Future directions

In conclusion, microtubule severing by KATANIN appears to be crucial for a broad range of important cellular, developmental, and physiological processes in plants.

Undoubtedly, studies on KATANIN brought about fascinating results on how microtubule severing can lead to the biased organization of cortical microtubules during conditional cell growth. It is fascinating to see that KATANIN provides a molecular connection between extracellular stimulation via physical signals such as light or mechanical forces and coordination of cell growth in a multicellular context. In this respect, it will be of interest to look on the microtubule counterpart and understand the features of the microtubule lattice at branching or crossover sites that are attracting KATANIN. Maybe these sites accumulate lattice defects (Davis et al., 2002; Díaz-Valencia et al., 2011; Jiang et al., 2017) or tubulin post-translational modifications which may target or regulate KATANIN activity similarly to SPASTIN (Lacroix et al., 2010; Sudo and Baas, 2010; Valenstein and Roll-Mecak, 2016). The field of plant KATANIN research is largely devoid of similar functional studies while data on modifiers of KATANIN activity and/or localization are scarce and mostly based on genetic evidence (e.g., Trehin et al., 2013; Wightman et al., 2013; Chen et al., 2014; Sassi et al., 2014). Therefore, *KATANIN* mutants should be better characterized regarding their biochemical and cell biological properties.

On the other hand, KATANIN function during mitotic cell division is still obscure and we believe that a very promising future direction will be to address in more detail the roles of microtubule severing in the PPB formation and maturation, in the mitotic spindle assembly and in the positioning and guidance of the phragmoplast. It is of particular interest to see how KATANIN is distributed within the PPB and how microtubule severing is coordinated during PPB narrowing since PPB maturation is significantly prolonged and frequently disturbed in *KATANIN* mutants (Panteris et al., 2011; Komis et al., 2017). It will be also necessary to decipher whether KATANIN is necessary (Panteris et al., 2011) or not (Komis et al., 2017) for mitotic spindle assembly and see whether KATANIN activity may be linked to spindle scaling issues as it does in animals (McNally et al., 2006; Loughlin et al., 2011). Finally, the role of KATANIN in the establishment of spindle—cell cortex association necessary for spindle positioning is another pending question.

Last but not least, is the necessity to characterize the importance of plant FIDGETIN and SPASTIN homologs in order to acquire a full view on the roles of microtubule severing proteins during plant growth and development.

## Author contributions

All authors listed have made a substantial, direct and intellectual contribution to the work, and approved it for publication.

### Conflict of interest statement

The authors declare that the research was conducted in the absence of any commercial or financial relationships that could be construed as a potential conflict of interest.
